# Selection Bias in Meta-Analyses of Gene-Disease Associations

**DOI:** 10.1371/journal.pmed.0020409

**Published:** 2005-11-22

**Authors:** Jin Ling Tang

## Abstract

Jin-Ling Tang discusses the issues arising from Ioannidis and colleagues' paper on biases in the Chinese genetic epidemiology literature, and possible solutions

Many studies have too small a sample size for their findings to be conclusive, but large studies are expensive and time-consuming. Meta-analysis is an alternative to conducting large studies in tackling the problem of small sample size, by combining available small studies to increase the total sample size. Since the 1980s, meta-analysis has been widely used in summarizing results from clinical trials of medical interventions and has also recently gained increasing attention in studying gene-disease associations. However, selection bias may occur in meta-analyses due to the inability to identify and include all conducted and relevant studies. Such selection bias can cause exaggerated or even false-positive gene-disease associations [1].

Failure to include all relevant studies is largely caused by selective publication of studies with certain results (publication bias), and the inability to identify studies published in languages other than English (language bias). Selection bias has been well recognized in meta-analyses of clinical trials [2–4]. Less is known about selection bias in meta-analyses of studies of gene-disease associations; such studies generally address weak associations and thus are particularly vulnerable to biases.

## A Study of the Chinese Literature

In their study published in this issue of *PLoS Medicine*, Pan and colleagues compared genetic studies conducted in mainland China with those from other places [5]. The researchers identified 12 gene-disease associations and compared a total of 161 Chinese studies and 309 non-Chinese studies. The Chinese studies were on average smaller in sample size than non-Chinese studies and appeared in the literature a few years after the first non-Chinese studies. Chinese studies in general reported a stronger gene-disease association and more frequently a statistically significant result. These two characteristics were more likely to occur in Chinese studies identified through PubMed than in those accessible only locally.

These findings suggest a variation or heterogeneity in the strength of the gene-disease association (often expressed in an odds ratio) observed between Chinese and non-Chinese studies. These studies are primarily case-control studies. Many factors may contribute to the variation in the estimate of odds ratio across such studies, such as the genetic make-up of the population studied, the type of patients included, the selection of controls, the quality of the study design, and the quality of the laboratory work. These factors could lead to either over- or under-estimation of the true odds ratio. However, it is difficult to conceive that any single factor, or combination of these factors, could consistently cause the exaggerated odds ratio in Chinese studies in all the topics (gene-disease associations) examined by Pan and colleagues. Selective publication is therefore a very likely and worrying explanation for their findings.

## Implications for Clinical Practice and Research

Selective publication can cause publication bias, which in turn could lead to false gene-disease associations in meta-analyses. It would be a disaster if a genetic screening program (in which healthy people are tested for a gene and offered a treatment if they test positive) were based on such a false association. Even if such a false gene-disease association were only subjected to further related investigations, this would be a waste of valuable resources for medical research.

Selective publication of positive studies in China and a few other Asian countries has been observed in clinical trials of acupuncture [6,7]. However, selective publication by no means exists in only the Chinese literature. It is probably a common phenomenon in the entire field of biomedical research. Given the fact that positive studies are more likely to be published than negative ones, and given the pressure on researchers worldwide to publish in indexed journals (especially in international journals with high impact factors), selective publication is likely to continue in the foreseeable future. As compared with English-speaking countries, selective publication is perhaps more likely to occur in non-English-speaking countries where there are a small number of indexed journals to publish local studies.

## Addressing the Problem

Journals accessible through PubMed or other major biomedical databases are unlikely to have the same mechanism of selection for publication as local journals that are less accessible to researchers outside the country, such as the Chinese journals. Thus, meta-analyses that include only internationally accessible studies (which is, currently, often the case for meta-analyses) are likely to have language or location bias. Meta-analyses that included only *local* studies could be even worse, as implied by Pan and colleagues' study. Inclusion of every study published worldwide would probably still not totally solve the problem, as many studies are never published or their publication is delayed. Odds ratios thus estimated would normally be an over-estimate.

Registration of studies is ideal and has been widely advocated for clinical trials [8]. Before such registration becomes universal practice, it would be important for journals, in selecting papers for publication, to emphasize the quality of the study rather than the size and direction of the odds ratio and the *p*-value of the statistical test. However, such an emphasis on quality (rather than the size and direction of the odds ratio) would not be much help to researchers who are currently doing meta-analyses.

Current researchers must strive to not only identify relevant studies but also examine the possibility of publication bias in the results. Although better tools have yet to be developed [9,10], current methods for detection and adjustment for publication bias in meta-analyses of clinical trials would be useful for meta-analyses of gene-disease associations [1,11,12].
